# Reconfigurable Disk-like Microswarm under a Sawtooth Magnetic Field

**DOI:** 10.3390/mi12121529

**Published:** 2021-12-09

**Authors:** Tao Zhang, Yuguo Deng, Bo Zhou, Jiayu Liu, Yufeng Su, Mu Li, Weiwei Zhang

**Affiliations:** 1 School of Mechanical Engineering, Zhengzhou University, Zhengzhou 450001, China; 202012202013874@gs.zzu.edu.cn (T.Z.); dyuguo3@gs.zzu.edu.cn (Y.D.); zhoubo3281@Outlook.com (B.Z.); JiayuLiu@stu.xjtu.edu.cn (J.L.); yufengsu@zzu.edu.cn (Y.S.); 2 Department of Pharmacy, The Second Affiliated Hospital of Harbin Medical University, Harbin 150086, China

**Keywords:** microrobot, disk-like swarm, sawtooth magnetic field, microassembly

## Abstract

Swarming robotic systems, which stem from insect swarms in nature, exhibit a high level of environmental adaptability and enhanced tasking capabilities for targeted delivery and micromanipulation. Here, we present a strategy that reconfigures paramagnetic nanoparticles into microswarms energized by a sawtooth magnetic field. A rotary-stepping magnetic-chain mechanism is proposed to address the forming principle of disk-like swarms. Based on programming the sawtooth field, the microswarm can perform reversible transformations between a disk, an ellipse and a ribbon, as well as splitting and merging. In addition, the swarms can be steered in any direction with excellent maneuverability and a high level of pattern stability. Under accurate manipulation of a magnetic microswarm, multiple microparts with complicated shapes were successfully combined into a complete assembly. This reconfigurable swarming microrobot may shed light on the understanding of complex morphological transformations in living systems and provide future practical applications of microfabrication and micromanipulation.

## 1. Introduction

Swarm behaviors in nature, such as bacterial colonies, flocks of birds and insect swarms [1,2,3], have attracted intensive research interest due to their unprecedented potentials in both fundamental research in living systems and technological applications [4,5,6,7,8,9,10,11,12]. Self-organized collective motion endows natural swarms with enhanced tasking capabilities, such as information communication, protection against invasion, higher foraging efficiency and low energy consumption, which cannot be completed by independent biological individuals.

Inspired by nature, artificial swarming microrobotic systems have been extensively reported in recent decades. These systems could be energized by an external magnetic field [5,13,14,15,16,17], chemical reaction [4,18,19,20,21], electric field [22,23,24], light [25,26,27,28,29,30] or ultrasound field [6,31,32,33]. Different interaction strategies have been designed to build micro/nanorobot swarms by emulating biological collaborative behaviors [34,35,36]. The various self-organizing microswarm patterns built by robotic scientists, such as liquid [17], chain [37,38], ribbon [39,40] and vortex [41,42,43,44], may help us to understand complex collective behaviors in living systems. Swarming robots have the advantages of the scale effect, robustness and deformation ability. Microswarms could generate the overall emergence of intelligence through the superposition of the number of robots, and it is possible for them to complete tasks that transcend their individual capabilities. Although part of a swarm of robots may be damaged and unable to work due to the disturbance of the external or internal environments, the remaining robots can maintain their original ability to ensure the completion of work tasks. Swarm robots can also change the shape or functional area of the swarm to interact with a complex environment. The construction of microrobot swarms could break through the limitations of a single microrobot in versatility and driving capability and provide a greater capacity for microfabrication and micromanipulation. However, the practical applications of precise microassembly using the swarming robotic system are still challenging. This may require us to further develop appropriate actuation strategies and microswarm patterns for more precise maneuverability and a higher level of pattern stability.

Modulation using external magnetic fields, which are inherently noncontact and chemical-fuel-free [45,46,47,48,49], may be a promising candidate. Various dynamic self-organization strategies have been successfully applied to trigger the formation of magnetic microswarms, such as alternating fields [14,16,38], rotating fields [5,44], conical magnetic fields [15,17] and so on. In this work, we designed a reconfigurable disk-like microswarm with excellent stability and maneuverability using a sawtooth magnetic field, which provides potential applications for precise microassembly. The magnetic microswarm could perform well-controlled and reversible transformations between the disk, ellipse and ribbon patterns, as well as splitting and reversible merging operations, by tuning the input parameter. The collective behaviors and the formation mechanism of the disk-like magnetic swarm are also investigated using molecular dynamics methods. Furthermore, the maneuverability of the swarms was exhibited by high-precision trajectory tracking. Finally, we demonstrate that the disk-like microswarm could manipulate multiple microparts with complicated shapes into a complete assembly. This may hold considerable promise for diverse future practical applications, ranging from microscale manipulation and assembly to biomedicine.

## 2. Materials and Methods

### 2.1. Experimental Setup

The assembly of superparamagnetic particles was achieved by generating a magnetic field using a pair of Helmholtz coils, which was controlled with sawtooth and continuous signal inputs, respectively. The control signals were generated by a PC, then amplified by a current amplifier to achieve an adequate field strength, up to 12 mT. The sample was enclosed by the 3D Helmholtz coils that were mounted on an optical microscope. Based on controlling the current inputs of the Helmholtz coils, the external sawtooth field and the uniform magnetic field could be generated in the workspace to build the microswarms in the desired patterns.

### 2.2. Materials

Superparamagnetic nanoparticles with a diameter of 800 nm were purchased from Aladdin (Fe_12_O_19_Sr, Aladdin, Shanghai, China). Before the magnetic actuation, the nanoparticles were initially subjected to an ultrasonic bath for 3 to 5 min. Firstly, magnetic nanoparticles were dispersed into a container (filled with deionized water) with a flat bottom and a glass cover. Then, the diffused particles were gathered by placing a permanent magnetic bead on the cover and were transferred into the workspace of the electromagnetic setup.

### 2.3. Computer Simulations

**Figure 1:**
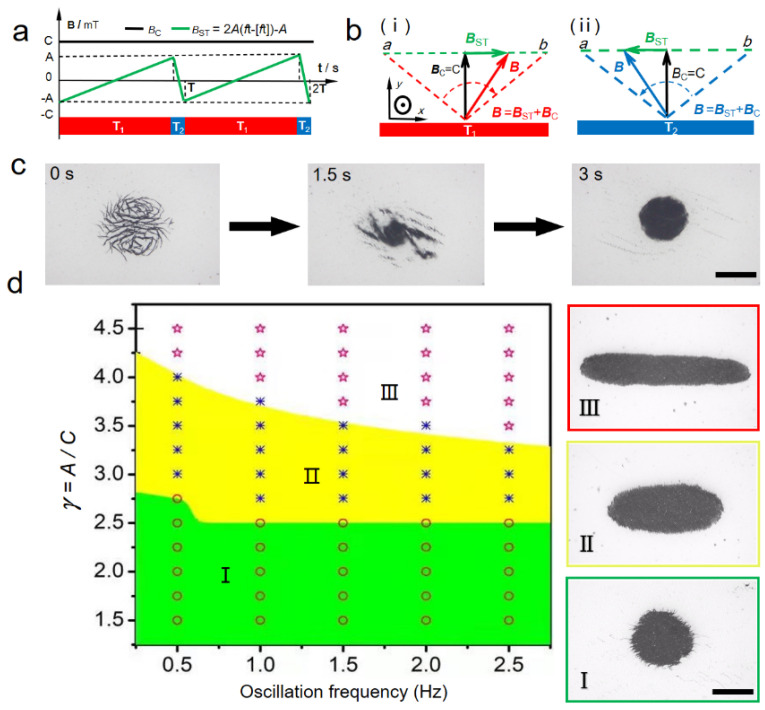
Formation of a disk-like microswarm using sawtooth magnetic fields. (**a**) The chart shows the wave forms of the sawtooth field, *B_ST_* (green), and the uniform field, *B_C_* (black). A complete period of the sawtooth wave comprises stages T_1_ and T_2_. (**b**) The schematics illustrate the synthesis principle of an oscillating magnetic field. The synthetic magnetic field, *B,* is superposed by the sawtooth field, *B_ST_*, and the perpendicular field, *B_C_*. (i) The synthetic field, *B* (red), swings forward slowly in T_1_. (ii) In stage T_2;_ the synthetic field, *B* (blue), wiggles back suddenly. (**c**) Nanoparticles are self-assembled into a disk-like microswarm in a short time. The scale bar is 350 μm. (**d**) The phase diagram presents three swarm patterns actuated by different magnetic fields. The disk-like swarm patterns can form in region (I). Under magnetic fields in region (II), the ellipse patterns are generated. The microswarms are elongated into ribbon-like microswarms under the fields in region (III). The scale bar is 300 μm.

All of the simulations were performed within the framework of the Large-scale Atomic/Molecular Massively Parallel Simulator (LAMMPS), which is a highly parallelized solver for molecular dynamics simulations [50]. The lattice Boltzmann (LB) method, which is an efficient and accurate method for modeling Newtonian flow [51], was adopted to deal with the Navier–Stokes equations. The LBM solver fix_lb_fluid was directly embedded into LAMMPS [52], where the fix was a kind of class offered by LAMMPS to apply external control on the simulation system. Each magnetic particle was treated as a sphere with a point dipole; the same method was used by S. Granick [15] and in our previous work [46]. Magnetic interactions were determined at each time step by solving the linear system of equations for each particle’s magnetic moment as a function of the field produced by the other particles and the spatially uniform, time-dependent external field, as shown in Figure 1a.

**Figure 2:**
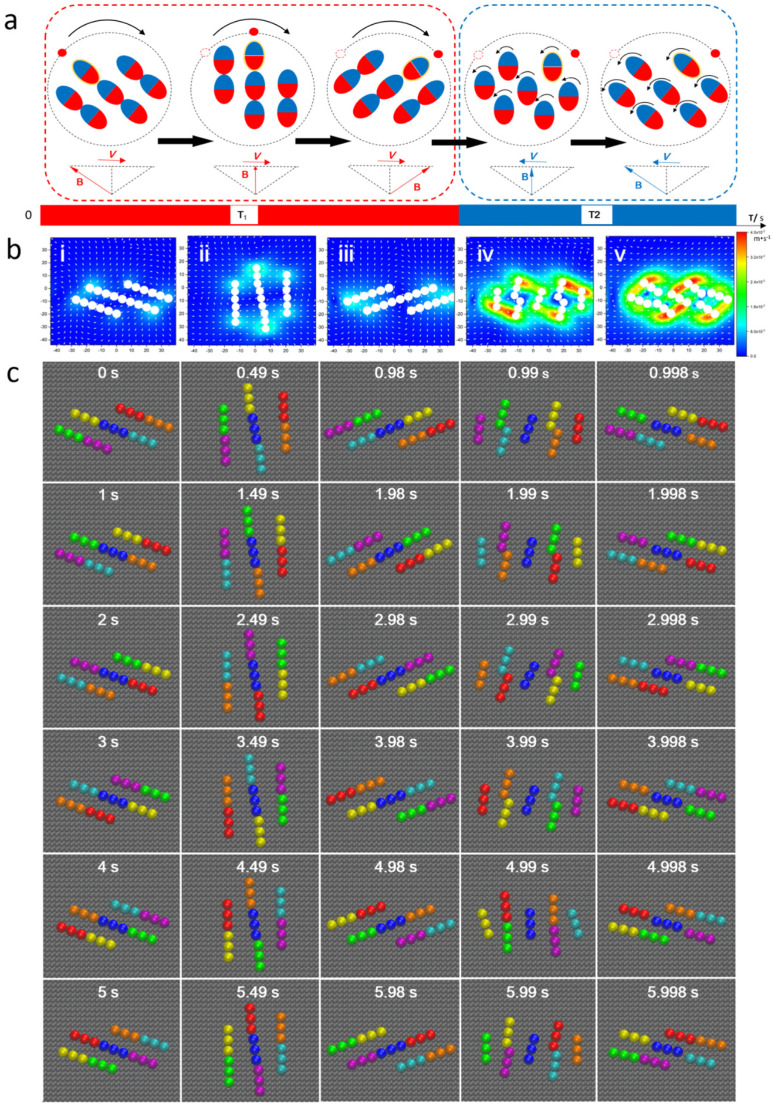
The formation mechanism analysis of a disk-like microswarm energized by a sawtooth magnetic field. (**a**) Schematic explanation of the rotary-stepping magnetic-chain mechanism. The ellipses represent tiny nanoparticle chains, and the parts in red and blue stand for the magnetic poles. (**b**) Simulation results of the flow field surrounding the magnetic chains actuated by a synthetic magnetic field with an amplitude ratio of *γ* = 3 and a frequency of *f* = 1 Hz. In stage T_1_, figures i–iii show the flow field with low velocity. Then, trimers rotate separately in stage T_2_, velocity in figures iv–v is much higher. (**c**) Dynamic sequence profile of a disk-like microswarm under a synthetic magnetic field. Trimers in diverse colors indicate different tiny magnetic chains.

The movement of the magnetic particles is captured by solving the equation that expresses Newton’s second law under the influence of both hydrodynamic forces and magnetic forces at a synthetic magnetic field with an amplitude ratio of *γ* = 3 and a frequency of *f* = 1 Hz. In order to clarify the formation mechanism of a disk-like microswarm energized by a sawtooth magnetic field, simulation analyses of fluidic fields and the collective dynamics of rigid magnetic chains were carried out and presented in Figure 2b,c.

## 3. Results and Discussion

### 3.1. Formation of a Disk-like Microswarm

By superposing a sawtooth magnetic field and a uniform magnetic field, we successfully reconfigured paramagnetic nanoparticles into a disk-like microswarm with a high level of pattern stability. In the *x* direction, the sawtooth magnetic field, *B_ST_*, was applied, with the condition of *B_ST_* = 2*A*(*ft* − [*ft*]) − *A*. As shown in Figure 1a, a complete period of the sawtooth wave comprises stages T_1_ and T_2_. The sawtooth field rose slowly at first in stage T_1_, then dropped rapidly in stage T_2_. In the *y* direction, a uniform magnetic field, *B_C_,* was applied, with a constant field strength of *C*. In the *xoy* plane, these two orthogonal magnetic fields were superposed into an oscillating magnetic field, *B*, as shown in Figure 1b. In stage T_1_, the resultant field, *B*, swung slowly from point *a* to point *b*, then waggled back rapidly in stage T_2_.

The formation process of the disk-like microswarm under a sawtooth magnetic field is demonstrated in Figure 1c and Video S1. Initially, paramagnetic nanoparticles formed uniformly distributed chain-like structures, and these tiny structures were the basis of disk-like microswarms. When the sawtooth magnetic field was applied, a small vortex of nanoparticles formed quickly in the center of these chains at *t* = 1.5 s. After collecting the surrounding nanoparticles together, a dynamic stable disk-like swarm was built at *t* = 3 s. Furthermore, the magnetic microswarm could perform well-controlled and reversible transformations between the disk-, ellipse- and ribbon-like patterns by tuning the input parameter. Figure 1d presents the relationship between the swarm patterns and the input magnetic fields with different frequencies, *f*, and amplitude ratios, *γ* = *A*/*C*. When *γ* was low, stable disk-like microswarms were formed in region I. As *γ* increased (region II), the ellipse-like pattern could be generated. Actuated by the fields in region III, nanoparticles self-organized into ribbon-like swarms.

Based on the molecular dynamics analysis of the forming process of the disk-like swarm under a sawtooth magnetic field, a rotary-stepping magnetic-chain mechanism was proposed, as shown in Figure 2a. The red and blue ellipses represent tiny chain-like structures self-organized from paramagnetic nanoparticles. In stage T_1_, these short chains attracted each other initially and formed three long chains. Then, the long chains rotated a specific angle, as a whole, when the complex field, *B*, gradually swung from point *a* to point *b* (Figure 1b(i)). Until the end of stage T_1_, the tiny chains at both ends of the long chains traveled a specific distance. When the field, *B*, swung back abruptly at the beginning of stage T_2_, the long chains were instantly disconnected into tiny chains again. Meanwhile, each tiny chain individually rotated back in place. In the following stage, T_1_, these tiny chains repeatedly combined into three new long chains, and the new long chains further rotated a specific angle based on the previous position.

Fluidic interaction is another critical factor for triggering the formation of disk-like microswarms. Magnetic chains experience a drag force due to the difference in velocity between particles and the surrounding fluids. The fluidic velocity field of the swinging magnetic chains actuated by a sawtooth field was simulated and analyzed. Figure 2b(i–iii) presents that the maximum flow velocity surrounded both ends of the magnetic chains throughout the whole stage of T_1_., and the maximal magnitude of flow velocity was relatively smaller than that in stage T_2_ due to the sudden backswing of field *B*. This indicates that the long chains suffered a relatively stronger fluidic resistance in stage T_2_, which leads to the disconnection of the long chains, as shown in Figure 2b(iv,v). The collective motion of the magnetic swarm was also investigated using molecular dynamics methods. Figure 2c exhibits that a disk-like swarm rotated 360 degrees, in steps, within six periods under a sawtooth magnetic field. At *t* = 0 s, seven tiny chains in different colors attracted each other into three long chains. During the first period, three long chains initially rotated slowly until *t* = 0.98 s, then disconnected into separate chains. Meanwhile, the external chains, such as the golden one (Figure 2c), moved a specific distance at first, then swung back individually, without translational displacement. As a result, the disk-like swarm rotated a fixed step angle. Three new long chains were formed at *t* = 1 s, and then the swarm repeated another rotational step. After six periods, the swarm realized a complete rotation (Video S2).

In order to compare the swarms presented here with traditional ‘vortex’ swarms, simulations were also carried out to generate swarms under rotating fields, as shown in Figure S1 and Video S3. The results demonstrate that the tiny chains of the microswarm rotated separately. It was quite different from the swarms under sawtooth magnetic fields, of which tiny chains were mostly connected to each other and rotating as a whole. This suggests that sawtooth magnetic fields might provide a higher level of pattern stability for disk-like swarms and offer a promising candidate for microassembly applications.

### 3.2. Controllable Transformation of Swarm Pattern

**Figure 3:**
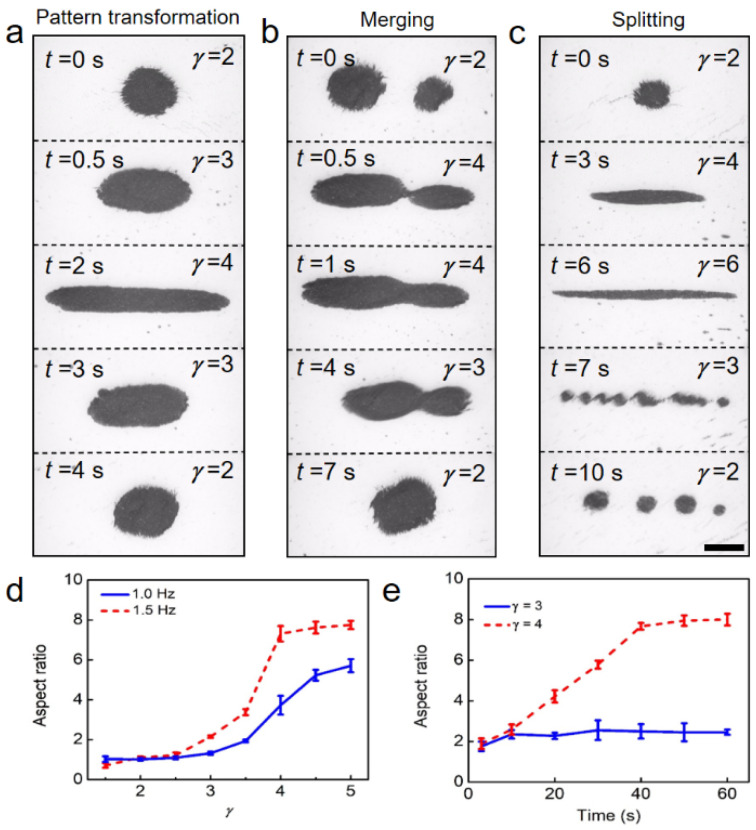
Controllable transformation of the swarm pattern. (**a**) Reversible transformations between disk, ellipse and ribbon patterns. (**b**) Merging process of two microswarms. (**c**) Controllable splitting of a magnetic microswarm. The scale bar is 400 μm. (**d**) Variations of the aspect ratio, *α,* with the amplitude ratio, *γ*. (**e**) Time evolution of the aspect ratio of magnetic microswarms. The applied sawtooth field frequency, *f*, is 1.5 Hz. In (**d**,**e**), each data point represents the average of 3 experiments. The error bars indicate the standard deviations.

The dynamic microswarm was capable of performing reversible anisotropic deformations, as well as controlled splitting and merging. Figure 3a and Video S4 demonstrate the transformation process of the swarm patterns. At *t* = 0 s, a disk-like microswarm was generated by the field of *γ* = 2 and *A* = 12 mT. With the increase in *γ*, the microswarm elongated and transformed into a stable ellipse-like swarm pattern at *t* = 0.5 s. When *γ* was further increased to 4, the microswarm evolved into the ribbon pattern. As *γ* was subsequently reduced, the microswarm shrank continuously and recovered to a disk pattern at *t* = 4 s.

Furthermore, the flexibility of the magnetic swarm could be utilized to merge separated microswarms (Video S5). Figure 3b presents the merging process of two swarms. Initially, two remote swarms were generated. When *γ* increased to 4, the two swarms elongated and started to contact at 0.5 s. Then, the two swarms merged into a ribbon-like swarm at *t* = 1 s. As *γ* decreased to 2, the ribbon-like swarm shrank slowly and finally became a disk-like swarm. In addition, swarm splitting can also be realized by tuning the input parameter *γ*. Initially, we built a long and thin swarm at *γ* = 5, as shown in Figure 3c and Video S6. After a quick drop of *γ* to 3, the swarm split into several small clusters. Finally, by turning *γ* to 2, four independent microswarms were obtained.

Figure 3d shows the relationship between the aspect ratio, *α,* of the microswarms and the amplitude ratio, *γ*. At a relatively low *γ*, the swarm remained in a disk pattern with *α* ≈ 1. Then, the curve rose as *γ* exceeded the critical value of 2.5. This suggests that the swarm started to transform into an ellipse pattern. In the end, the curve reached a plateau stage where the ratio, α, did not increase with *γ*. Moreover, the largest aspect ratio of the swarms increased with the field frequency, as shown in Figure 3d. The transfer efficiency of the swarm patterns was also investigated. The disk-to-ellipse transformation of swarm pattern could be achieved within 10 s (Figure 3e, *γ* = 3). However, the generation of ribbon-like swarms required more time to reconfigure the magnetic nanoparticles. The aspect ratio, *α,* increased steadily with time until *t* = 40 s, when the transformation of a ribbon-like swarm was realized (Figure 3e, *γ* = 4).

### 3.3. Practical Applications of Micromanipulation and Microassembly

**Figure 4:**
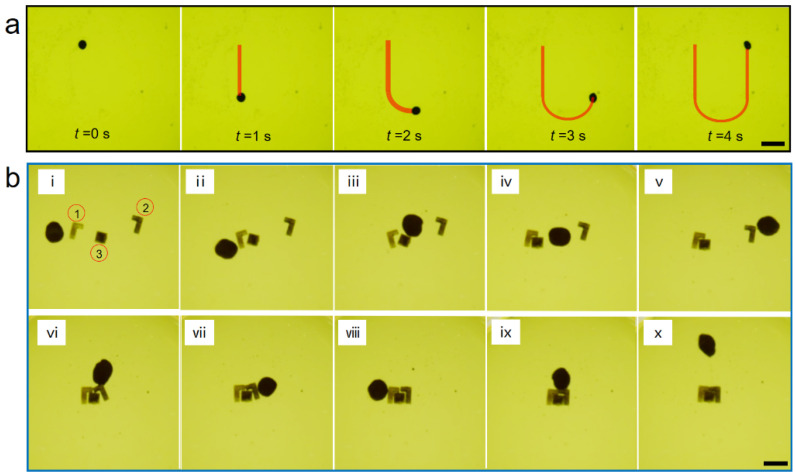
Trajectory manipulation of a magnetic microswarm and the precise microassembly of ‘Tetris’ microparts. (**a**) The disk-like microswarm under the programming magnetic fields. The scale bar is 800 μm. (**b**) A complete assembly of the distributed ‘Tetris’ microparts under the manipulation of a swarm. Figures i–vii show that the three blocks are preliminarily assembled. Then, in figures viii–x, the small gaps between blocks are eliminated by pushing the assembly gently on the left and top sides. The scale bar is 1 mm.

Compared with the traditional actuation strategies of a ‘vortex’ swarm under a rotating magnetic field, the swarm energized by a sawtooth field exhibited excellent maneuverability and pattern stability, which could provide more facility for the practical applications of micromanipulation and microfabrication. The maneuverability of the swarms was exhibited by high-precision trajectory tracking (Video S7). Figure 4a illustrates the sequence profiles of a disk-like microswarm traveling along a U-path trajectory under programming magnetic fields. The swarms could be steered in different directions by simply adjusting the direction of the centerline of the swing field, *B* (Figure 1b). The swarm advanced straight at first, then took a U-turn to move backwards. The trajectory fit well with the U-path, which means that the swarm’s direction of motion could be controlled continuously. Finally, the disk-like microswarm was applied to a precise microassembly practice. Three ‘Tetris’ blocks were scattered randomly at first, as shown in Figure 4b and Video S8. The microswarm was manipulated to carry block 1 close to block 3 (Figure 4b(ii)). Then, the orientation of block 3 was adjusted slightly to face the opening of block 1 for further assembly (Figure 4b(iii,iv)). Next, block 2 was shifted adjacent to block 3 (Figure 4b(v,vi)). After fine-tuning the long leg of block 2, the three blocks were preliminarily assembled (Figure 4b(vii)). To eliminate small gaps between these blocks, the swarm was manipulated to push the assembly quite gently on the left and top sides (Figure 4b(viii,ix)). Under accurate manipulation of the magnetic microswarm, the three ‘Tetris’ microparts with complicated shapes were successfully combined into a complete ‘square’ assembly (Figure 4b(x)).

## 4. Conclusions

By superposing a sawtooth magnetic field and a uniform magnetic field, we successfully reconfigured paramagnetic nanoparticles into a disk-like dynamic microswarm with a high level of pattern stability. The dynamic microswarm was capable of performing reversible anisotropic deformation, as well as controlled splitting and merging. Based on the molecular dynamics analysis of the forming process of a disk-like swarm under a sawtooth magnetic field, a rotary-stepping magnetic-chain mechanism was proposed. The maneuverability of the swarms was exhibited by high-precision ‘U’ trajectory tracking. Compared with the traditional actuation strategies of ‘vortex’ swarms, with separately rotating magnetic chains, the tiny chains of the disk-like swarm under a sawtooth magnetic field are mostly connected to each other and rotating as a whole. This suggests that a sawtooth magnetic field could enhance the pattern stability of disk-like swarms, which provides a promising candidate for microassembly applications. In order to verify the applicability of the disk-like swarms reported here, a ‘Tetris’ assembly experiment was also carried out. Under accurate manipulation the of magnetic microswarm, several ‘Tetris’ microparts with complicated shapes were successfully combined into a complete ‘square’ assembly. This reconfigurable microrobot swarm may shed light on the understanding of complex morphological transformations in living systems and holds considerable promise for the future practical applications of microfabrication and micromanipulation.

## Data Availability

All data needed to evaluate the conclusions in the paper are present in the paper and/or the Supplementary Materials. Additional data related to this paper may be requested from the authors.

## Supplementary Materials

The following are available online at https://www.mdpi.com/article/10.3390/mi12121529/s1, Figure S1: Dynamic sequence profile of disk-like swarm under rotating magnetic field (*f* = 1 Hz). Trimers in diverse colors indicate different tiny magnetic chains. Video S1: The formation of a disk-like swarm generated by a sawtooth magnetic field. Video S2: Simulation of the forming process of a disk-like swarm under a sawtooth magnetic field. Video S3: Simulation of the forming process of a disk-like swarm under a rotating magnetic field. Video S4: Reversible transformations between disk, ellipse and ribbon patterns of the swarm. Video S5: Merging process of two swarms. Video S6: Splitting one swarm into four smaller swarms. Video S7: Trajectory tracking of a disk-like swarm. Video S8: The assembly of distributed ‘Tetris’ microparts using a sawtooth magnetic swarm.
