# Supplementary material for: Reconfigurable Disk-like Microswarm under a Sawtooth Magnetic Field
Source: Micromachines (Basel). 2021 Dec 9;12(12):1529. doi: 10.3390/mi12121529 (PMC8708609; doi:10.3390/mi12121529)
Supplement: Supplementary file 1 [file micromachines-12-01529-s001.zip › Supplementary information.pdf]

micromachines

MDPI

# Supplementary Information for "Reconfigurable disk-like microswarm under a sawtooth magnetic field"

**Tao Zhang [1], Yuguo Deng [1], Bo Zhou [1], Jiayu Liu [1], Yufeng Su [1], Mu Li [2,*], Weiwei Zhang [1,*]**

[1] School of Mechanical Engineering, Zhengzhou University, Zhengzhou, 450001, China.
[2] Department of Pharmacy, Department of Radiology, the Second Affiliated Hospital of Harbin Medical University, Harbin, China.
* Correspondence: limu@hrbmu.edu.cn (M. L.); vivid@zzu.edu.cn (W. Z.)

## *1. The forming process of swarms generated by rotating magnetic field*

Simulations were also carried out to generate disk-like swarms under rotating magnetic field. Figure S1 demonstrates sequence profiles of microswarm energized by a rotating magnetic field. Initially, tiny magnetic chains are attracted closely and form a disk-like swarm under the combined actions of magnetic force and fluid interaction. Then, each chain, like the golden one, rotates continuously around its own axis under the action of external rotating magnetic field (Video S3). Meanwhile, the external chains revolves around the central chain as shown in Figure S1. However, the rotation of individual chains does not keep pace with the revolution. As a result, the swarm rotates only a quarter of circle at the end of the period. Four periods are required to finish a complete rotation of the microswarm.

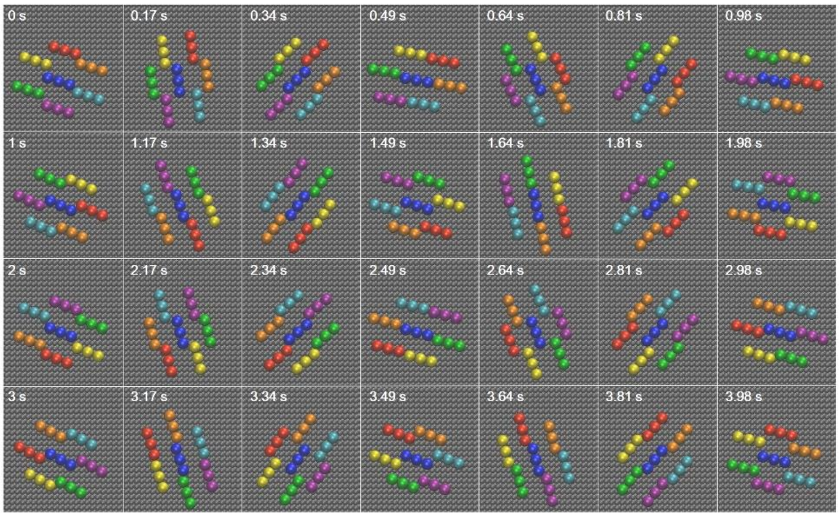


Figure *S*1 Dynamic sequence profile of disk-like swarm under rotating magnetic field ($f$ = 1 Hz). Trimers in diverse colors indicate different tiny magnetic chains.

Under the actuation of a rotating magnetic field, tiny chains of microswarm rotates separately. It is quite different from swarms under sawtooth magnetic fields, of which tiny chains are mostly

connected to each other and rotate as a whole. This suggests that sawtooth magnetic field might provide higher pattern stability for disk-like swarm.
